# Enhancement of Cetuximab-Induced Radiosensitization by JAK-1 Inhibition

**DOI:** 10.1186/s12885-015-1679-x

**Published:** 2015-10-12

**Authors:** James A. Bonner, Hoa Q. Trummell, Andrew B. Bonner, Christopher D. Willey, Markus Bredel, Eddy S. Yang

**Affiliations:** The University of Alabama at Birmingham, Department of Radiation Oncology, Hazelrig-Salter Radiation Oncology Center, Suite 2262, 1700 6th Avenue South, Birmingham, AL 35249 UK

## Abstract

**Background:**

It is known that cetuximab (an epidermal growth factor receptor [EGFr] inhibitor) is a radiosensitizer. Also, cetuximab is known to only partially inhibit the signal transducer and activator of transcription – 3 (STAT-3); a mediator of protection from apoptosis. Studies were performed to determine if the radiosensitizing effects of cetuximab could be enhanced with the addition of an inhibitor of STAT-3.

**Methods/Results:**

The interaction of JAK-STAT-3 inhibition ([JAK1i]; Calbiochem, LaJolla, CA) and EGFr inhibition (cetuximab) was assessed with and without radiation. Four human head and neck cell lines were studied: UM-SCC-1 and UM-SCC-5, and two modified UM-SCC-5 lines; a STAT-3 knockdown line (STAT-3-2.4) and control (NEG-4.17). Exposure to either 0.5 μg/ml of cetuximab or 1 μM JAK1i for 8 or 24 h resulted in reduced activated STAT-3 (immunoblot), and the combination treatment showed greater reduction in activated STAT-3 compared to the individual treatments. The use of either post-radiation JAK1i (1 μM for 72 h) or post-radiation cetuximab (0.5 μg/ml) enhanced radiation-induced anti-proliferative and apoptotic effects but the greatest enhancement was seen when cells were exposed to both JAK1i and cetuximab post-radiation. Similar results were seen for radiosensitization as assessed by colony formation. Finally, the combination treatment of JAK1i (1 μM) and cetuximab (0.5 μg/ml), following radiation, resulted in an increase of unrepaired radiation-induced DNA double strand breaks at 6 and 24 h after radiation compared to the use of post-radiation JAK1i or cetuximab alone as delineated by neutral comet assay.

**Conclusions:**

These findings suggest that dual inhibition of EGFr (cetuximab) and JAK-STAT-3 (JAK1i) leads to greater radiosensitization than with either cetuximab or JAK1i alone and suggests that this combination treatment may be clinically relevant even for tumors with a marked range of STAT-3 activity.

## Background

Cetuximab is an inhibitor of the Epidermal Growth Factor Receptor (EGFr) that binds to the EGFr ligand binding domain, thereby inhibiting downstream EGFr signaling involved in cellular growth [1]. In the clinic, cetuximab has shown modest activity as a single agent for metastatic head and neck cancer (13 % response rate when used alone for recurrent disease) and radiosensitizing activity for locoregionally advanced head and neck cancer [2–4]. Since the EGFr signaling pathway involves multiple downstream phosphorylation reactions and crosstalk with other signaling pathways, it is possible that the anti-tumor effects of EGFr inhibition can be enhanced by inhibiting other downstream effectors of EGFr signaling.

The signal transducer and activator of transcription-3 (STAT-3) is a protein that lies downstream of EGFr and activation of EGFr leads to activated STAT-3, which in turn protects cells from apoptosis. However, it is also known that several other signaling events activate STAT-3. The Janus Kinases (JAK1 and JAK2) are important activators of STAT-3. Furthermore, other signaling cascades, such as the SRC pathway, can activate the JAK/STAT-3 cascade [5]. We have previously shown that cetuximab-induced inhibition of EGFr leads to inhibition of activated STAT-3, but this inhibition is incomplete [1]. It is likely that other activators of STAT-3, such as the Janus Kinases, circumvent more complete STAT-3 inhibition when cetuximab is used alone. Therefore, it is believed that STAT-3 continues to affect downstream protection from apoptosis, and other STAT-3 mediated events such as angiogenesis, even when it is partially inhibited by cetuximab.

In an effort to achieve more complete inhibition of EGFr signaling, we explored the combined inhibition of EGFr and JAK–STAT-3 (dual inhibition) with and without radiation in human head and neck squamous cell cancer cell lines with a variety of STAT-3 expression. One of the tested cell lines had partial knockdown of STAT-3 as previously described [6, 7]. It was determined that JAK-STAT-3 inhibition accentuated the radiosensitizing properties of cetuximab in all cell lines. Although we initially set out to determine whether JAK1i increased the known cetuximab-induced radiosensitizing properties, we found that both agents were radiosensitizers and the radiosensitizing effects were greatest when the agents were given together.

## Methods

### Cell culture

Human head and squamous cell cancer cell lines were grown in Dulbecco’s Modified Eagle’s Medium containing 10 % heat-inactive fetal bovine serum supplemented with 2 μM L-glutamine and incubated in a humidified chamber at 37 °C with 5 % CO^2^ as previously described [6, 7]. UM-SCC-1 and UM-SCC-5 were obtained from Dr. Thomas Carey at the University of Michigan. UM-SCC-5 cells were used to create stable transfectants of a short hairpin RNA against STAT-3 (STAT-3-2.4 cells). These cells were created by transfecting UM-SCC-5 cells with a pBABE-U6 vector containing STAT-3 short hairpin RNA (shRNA) as previously described [6]. Following transfection, the STAT-3-2.4 cells showed approximately 50 % STAT-3 knockdown as previously described [6, 7]. Control cells were also previously created by transfection with a mutated or negative STAT-3 shRNA form of the short hairpin RNA against STAT-3 (NEG 4.17 cells) and do not show significant STAT-3 knockdown [7].

### Immunoblots

Immunoblots were used to analyze the protein expression levels of STAT3 as previously described [6, 7]. UM-SCC-1, −5 and transfected STAT3-2.4 and NEG4.17 cells were assessed for STAT-3, phosphorylated STAT-3 and GAPDH. Cell lysates were prepared and equal amounts of protein were loaded in each gel lane. Separation was performed by 10 % sodium dodecyl sulfate-polyacrylamide gel electrophoresis (SDS–PAGE) and transferred to Immobilon-P membrane (Millipore Corp, Bedford, MA). The immunoblots were blocked in 10 % milk–Tris-buffered saline with Tween-20 (TBS-T) (20 mmol/L Tris HCL [pH 7.5], NaCl 137 mmol/L, and 0.05 % Tween-20) for 1 h at room temperature. The primary antibodies of anti-STAT-3, anti-p-STAT-3 (Cell Signaling Technologies, Beverly, MA), anti-EGFr (Sigma-Aldrich, St. Louis, MO), anti-p-EGFr (cell signaling technologies, Beverly, MA) and anti-GAPDH (Santa Cruz Technologies, Inc., Santa Cruz, CA) were incubated overnight at 4 ° C with 2 % milk–TBS-T. The secondary antibody, anti-mouse–IgG–horseradish peroxidase antibody (Sigma Chemical Company, St. Louis, MO) was incubated at room temperature for 1 h. The blots were developed by chemiluminescence (Amersham Life Sciences, Inc., Arlington Heights, IL).

### Cell proliferation

The effects of various treatments on cell proliferation were assessed by a cell proliferation assay as previously described [6, 7]. Briefly, UM-SCC-1, −5, and the transfected STAT3-2.4 and NEG 4.17 cells were plated in a manner to allow multiple days of proliferation. Following the entry of cells into an exponential growth phase, they were exposed to various combinations of cetuximab (0.5 μg/ml), 1 μM Janus Kinase inhibitor ([JAK1i] Calbiochem, LaJolla, CA) and/or radiation (2 Gy). The cells were assessed for cell counts every 24 h. The cells were removed from plates with trypsin and counted using a cell counter (Beckman Coulter, Fullerton, CA). Illustrative experiments demonstrate results following 72 h of growth.

### Apoptosis

Apoptotic cell death following exposure to various treatments was quantified by using the Annexin V-FITC apoptosis detection kit (BioVision Research Products, Mountain View, CA) as previously described [8]. UM-SCC-1, −5, and the transfected STAT3-2.4 and NEG 4.17 cells were treated with various combinations of cetuximab (0.5 μg/ml), JAK1i (1 μM) and/or radiation (2 Gy) in a manner identical to the cell proliferation assay. Following 72 h of incubation, the cells were isolated and the data were collected using a Becton Dickinson FACScan system. This information was analyzed using CellQuest v3.1 software (Becton Dickinson, San Jose, CA).

### Colony formation

Cell survival was assessed by colony formation as previously described [6, 7]. Briefly, cells were plated the day before the start of the experiment in order to allow time for attachment of the cells. The cells were exposed to various doses of radiation, either alone or with exposure to JAK1i, cetuximab or both agents just prior to radiation and during the post-radiation period. The cells were allowed to form colonies over 12–14 days. They were subsequently fixed, stained and counted (colonies of > 50 cells) for colony formation as previously described.

### Neutral comet assay

The accumulation of DNA double strand breaks (dsbs) was assessed by the neutral comet assay as previous described [7]^.^ Briefly, exponentially growing UM-SCC-1, −5, and the transfected UM-STAT3-2.4 and NEG 4.17 cells were plated at a concentration of 150,000 cells in 60 mm2 tissue culture dishes. After a 16 h incubation in the presence of cetuximab (5 μg/ml), JAK1i (1 μM) or the combined treatment with or without radiation, the cells were scraped and processed for Neutral Comet Assay at various times following radiation as described by Trevigen (Gaithersberg, MD). Following the staining with SYBR Green I, the slides were viewed by fluorescence microscopy (Evos f1, Advanced Microscopy Group, Bothell, WA). Tail moment and percent DNA in the tail were analyzed using CometScore Freeware.

### Statistical analysis

All experiments were performed with at least three independent experiments. Representative immunoblots were selected for presentation. For experiments other than the immunoblots, results were expressed as the mean ± standard error (SE). For the cell proliferation and apoptosis assays, the statistical significance between treatment groups was determined using a two-way ANOVA assessment with software from GraphPad Prism, San Diego, California. Overall comparisons of certain conditions were made using comparisons of all four cell lines in a simultaneous two-way ANOVA assessment.

### Ethics

This research did not involve human subject.

## Results

**Fig. 1:**
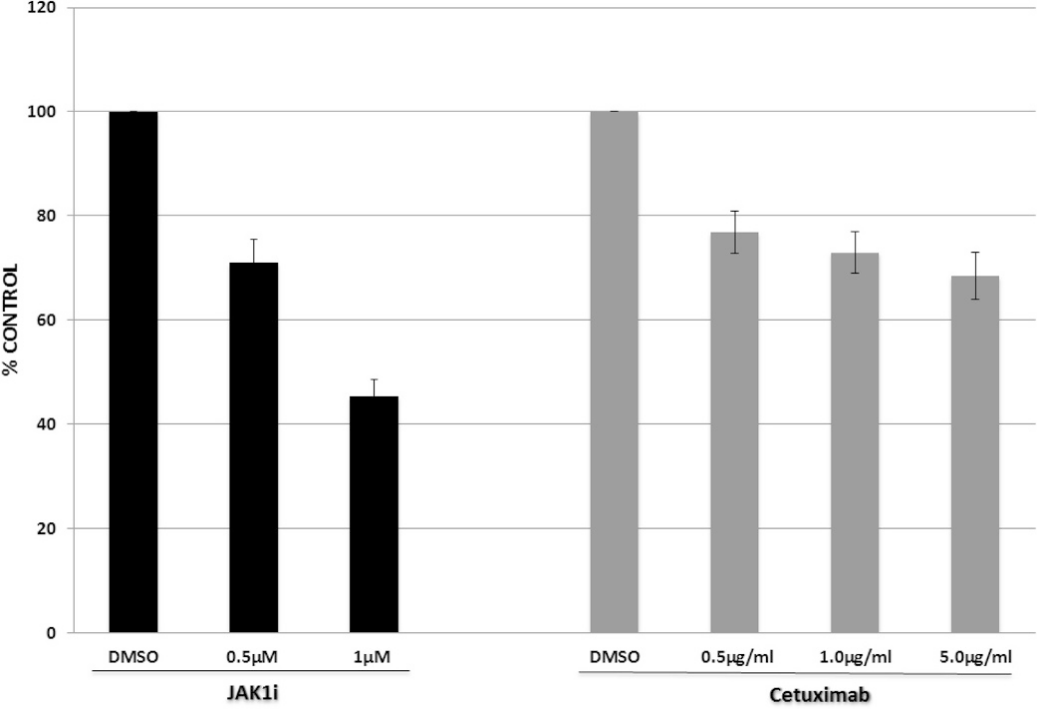
Concentration-response assessments were made for JAK1i and cetuximab in UM-SCC-1 cells as a representative cell line. Cells were exposed to various concentrations of the inhibitors for 72 h and subsequently assessed for proliferation as described in the methods section

Since cetuximab and JAK1i have previously been shown to have anti-proliferative properties [1, 9, 10], studies were performed to determine the concentration-response characteristics of these agents with respect to their anti-proliferative effects in human head and neck squamous cell carcinoma cell lines. Utilizing the UM-SCC-1 cells, we sought to find concentrations of these agents that resulted in less than 60 % reductions in proliferation after 72 h exposures. This minimal effective level was sought because subsequent experiments would involve combinations of the agents and it was felt that more effective exposures could blunt the meaningful examination of combining these agents. When UM-SCC-1 cells were treated with 5 μg/ml (or less) of cetuximab alone or 1 μM (or less) of JAK1i alone for 72 h, the resulting anti-proliferative effects were less than 60 % inhibition (Fig. 1). Similar results were found in the other studied cell lines. Therefore, these concentration ranges (for the two agents) were utilized for subsequent experiments. It is noteworthy that the subsequently studied combination effects varied somewhat with the concentrations of JAK1i or cetuximab (Figs. 3 and 4).

**Fig. 2:**
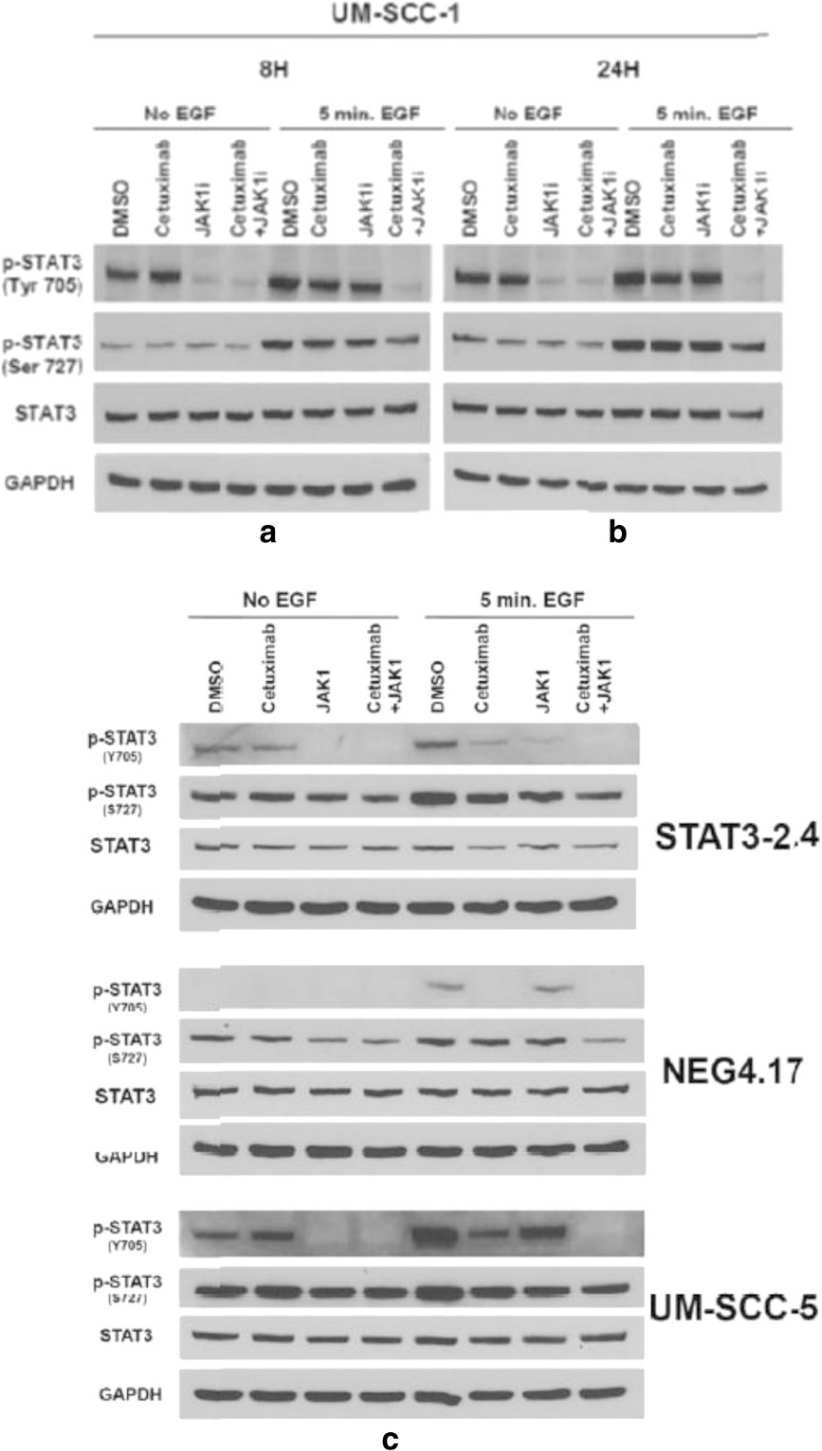
Immunoblot analyses revealed that the phosphophorylation of STAT-3 was significantly reduced with the dual treatment of cetuximab and JAK1i as compared to either individual agent alone. UM-SCC-1, UM-SCC-5, STAT-3 knockdown cells (STAT3-2.4) and control transfected cells (NEG4.17) were treated with cetuximab (5 μg/ml) and/or JAK1i (1 μM) for either 8 or 24 h with or without 5 mins of exposure to EGF (60 ng/ml) and subsequently assessed as described in the methods section. Protein lysates were subjected to SDS-PAGE and Western blot analysis for STAT-3, p-STAT-3(Tyr705) and p-STAT-3(Ser727). GAPDH was used to control for loading variability. Representative immunoblots for the UM-SCC-1 cells assessed at 8 h **a** and 24 h **b** are shown. Representative immunoblots for the remaining cell lines are shown for the 24 h time point **c**

**Fig. 3:**
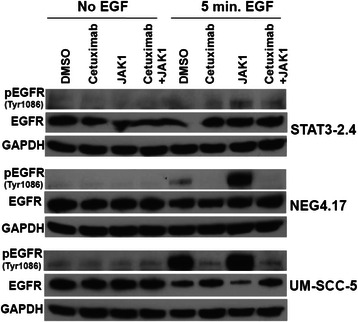
Immunoblot analyses revealed that cells treated with cetuximab (5 μg/ml) and the combination of cetuximab and JAK1i (1 μM) showed reductions in p-EGFr. Additionally, cells treated with JAK1i (1 μM) alone showed increased p-EGFr. Parental UM-SCC-5 cells, STAT-3 knockdown cells (STAT3-2.4) and control transfected cells (NEG 4.17) were treated with cetuximab (5 μg/ml) and/or JAK1i (1 μM) for 24 h (with and without 5 mins of exposure to EGF [60 mg/ml] at the end of the 24 h) and assessed for p-EGFr, EGFr and GAPDH as described in the methods section

Next, the assessment of EGFr inhibition (cetuximab), with or without the addition of JAK-STAT-3 inhibition (JAK1i), and its effect on STAT-3 protein expression in four human head and neck cancer cell lines was studied. The STAT-3 knockdown cells (STAT 3–2.4) previously demonstrated approximately 45–55 % less STAT-3 protein compared to the control cells (NEG 4.17) [6, 7]. Immunoblots were used to assess the effects of various treatments on overall STAT-3, activated STAT-3 (primarily p-STAT-3 [Tyr 705] and to a lesser extent p-STAT-3 [Ser 727]). The immunoblots were performed with and without 5 mins of EGF stimulation at the end of each treatment (Fig. 2). For the UM-SCC-1 cells, STAT-3 protein was assessed at 8 and 24 h following exposure to cetuximab alone (0.5 μg/ml), JAK1i (1 μM) alone or cetuximab and JAK1i. The remaining cell lines were assessed after 24 h exposures to the agents (Fig. 2c). The UM-SCC-1 cells showed a suggestion of a decrement in overall STAT-3 with the combination treatment (JAK1i and cetuximab) at 8 and 24 h (Fig. 2a and b), but the other cell lines did not show consistent reductions in overall STAT-3 with these treatments (Fig. 2c). However, activated STAT-3 was decreased with either cetuximab or JAK1i and the greatest decrement in activated STAT-3 was generally seen for the combination of cetuximab and JAK1i. Figure 2c also demonstrates the decreased STAT-3 levels in the STAT-3 knockdown cells (STAT 3–2.4) compared to control (NEG 4.17) or parental a (UM-SCC-5) cells. The latter three cell lines were also assessed for EGFr and p-EGFr (Tyr 1086) following the same 24 h exposures (noted above for p-STAT-3 assessments) to the two inhibitors (Fig. 3). Cetuximab, with or without JAK1i resulted in decreased p-EGFr but JAK1i alone caused an increase in p-EGFr possibly due to a feedback mechanism.

**Fig. 4:**
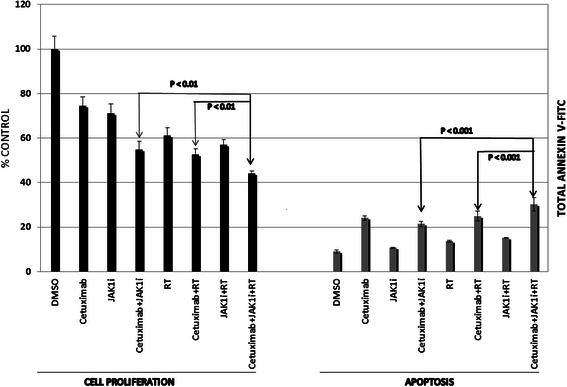
The addition of JAK1i (0.5 μM) to cetuximab and radiation resulted in an enhancement of radiation-induced anti-proliferative (left) and apoptosis effects (right) in UM-SCC-1 cells. Cells were treated for 72 h with cetuximab (0.5 μM) with and without radiation (2 Gy) and subsequently assessed for cell proliferation or apoptosis as described in the methods section

Since the immunoblot results demonstrated enhanced inhibition of p-STAT-3 for the combination of JAK1i and cetuximab, studies were performed to determine if the combination of the two agents resulted in a greater impact on radiation-induced anti-proliferation or apoptotic effects compared to treatments with the individual agents. These studies were building on previous studies in which STAT-3 knockdown was found to accentuate the effects of radiation [6]. The UM-SCC-1 cells were assessed for proliferation changes and apoptosis following exposure to JAK1i alone (0.5 μM), cetuximab alone (0.5 μg/ml), the combination of the two inhibitors, each inhibitor with radiation (2 Gy) or the combination of inhibitors with radiation (Fig. 4). Each individual inhibitor accentuated the anti-proliferative effects of radiation in UM-SCC-1 cells (Fig. 4). Furthermore, when JAK1i was added to the combination treatment of cetuximab and radiation, there was an enhancement of the anti-proliferative effects compared to cetuximab and radiation without JAK1i. Also, there was a significantly greater effect regarding the inhibition of proliferation when radiation was combined with the two inhibitors compared to the two inhibitors without radiation. These findings were mirrored by studies examining apoptosis for the UM-SCC-1 cells (Fig. 4). The addition of JAK1i to cetuximab and radiation resulted in greater apoptosis, that was statistically significant, compared to cetuximab and radiation without JAK1i (Fig. 4). Additionally, apoptosis was significantly greater for the two inhibitors in combination with radiation compared with the two inhibitors without radiation (Fig. 4). Therefore, the dual inhibition of STAT-3 and EGFr showed greater anti-proliferative effects and apoptosis compared to the use of either single inhibitor and dual inhibition accentuated radiation-induced anti-proliferative effects and apoptosis to a greater extent compared to either single inhibitor in the UM-SCC-1 cells.

**Fig. 5:**
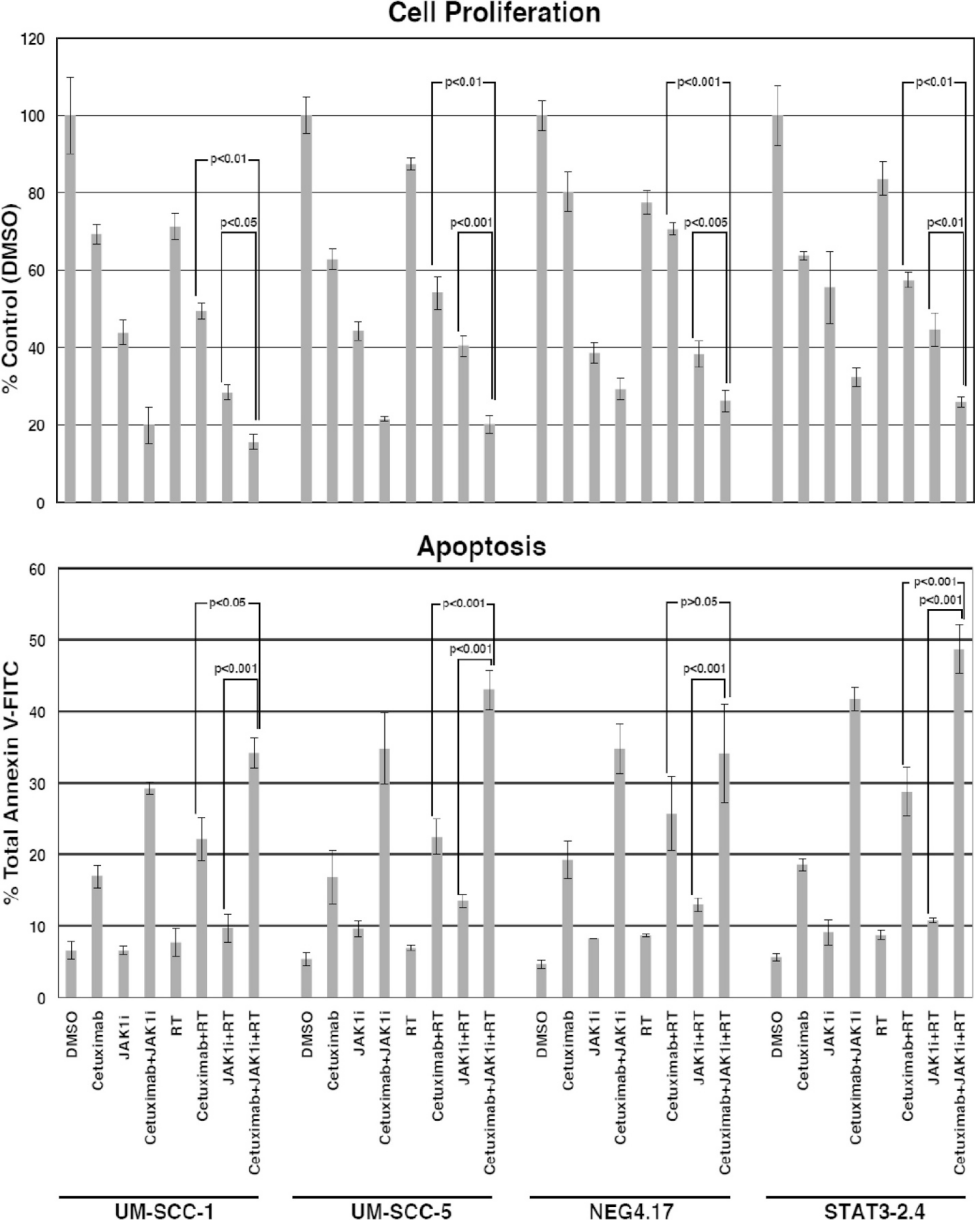
The use of JAK1i (1 μM) increased the effects of cetuximab (0.5 μg/ml) in all four cell lines when cell proliferation or apoptosis was assessed. The data represent the mean and standard error of three assessments. *Top*. Cell proliferation was assessed 72 h after indicated treatments and normalized to vehicle-treated cells (100 %). *Bottom*. Apoptosis was assessed in exponentially growing cells that were collected after 72 h treatments and analyzed using Annexin V-FICT kit

Next, additional cell lines were studied to test whether these results were potentially generalizable. Cell lines with various levels of STAT-3 were assessed. Cell proliferation assessments were undertaken for all four cell lines under similar conditions (slightly different JAK1i concentration) as noted above for the UM-SCC-1 cells. Greater decrements in cell proliferation were noted following exposure to both agents (JAK1i, [1 μM] and cetuximab [0.5 μg/ml] for 72 h), compared to either agent alone in these additional cell lines (Fig. 5). The various cell lines showed slightly different sensitivities to cetuximab alone or JAK1i alone. Compared to the previous experiments with UM-SCC-1 (Fig. 4), these studies were undertaken with a slightly higher concentration of JAK1i (1 μM) that interacted substantially with both cetuximab and radiation. The overall significance (taking into account all four cell lines) of adding JAK1i to cetuximab and RT [*p* = 0.0001] was pronounced. Likewise, there was a significant effect for the addition of cetuximab to JAK1i and RT [*p* = 0.0001]. However, when the higher concentrations of JAK1i (1 μM) were utilized (compared to early experiments [Fig. 4]), there was a more pronounced interaction between JAK1i and cetuximab. Therefore, there was only a suggestion that the overall inhibition of proliferation for radiation with the two inhibitors was greater compared to the two inhibitors without radiation. Additionally, when the four cell lines were assessed individually, the use of combined cetuximab/JAK1i resulted in significantly enhanced radiation-induced anti-proliferation compared to the use of either agent alone (Fig. 5). Although these effects of treatment with cetuximab, JAK1i and radiation were consistent with the immunoblot data in Fig. 2, some cell lines showed greater inhibitory effects with immunoblot data which did not directly correlate with greater effects regarding the anti-proliferative effects. These findings suggested that EGFr and STAT-3 were just two of many factors affecting radiosensitivity.

Since STAT-3 is known to protect cells from apoptosis, the above noted treatment-related effects on cell proliferation were further assessed to determine if they correlated with apoptotic events. Cetuximab and/or JAK1i were tested with and without radiation (Fig. 5). The results correlated with the cell proliferation findings. As with the cell proliferation studies, greater apoptosis was seen when JAK1i was added to the combination of cetuximab and radiation. The overall levels of significance for the comparisons of cetuximab/JAK1i with RT *vs*. either cetuximab with RT [*p* = 0.0001], or JAK1i with RT [*p* = 0.0001], were pronounced. These effects were statistically significant for all individual cell lines except the comparison of cetuximab/JAK1i with RT *vs*. cetuximab and RT in the control transfectants (NEG 4.17 cells). As with the proliferation studies, there was a suggestion that cetuximab/JAK1i and RT produced more apoptosis than cetuximab/JAK1i, but it was not significant [*p* > 0.05]. Once again, this comparison was significant when cells were exposed to lower concentrations of JAK1i (0.5 μM) as shown in Fig. 4. In summary, these findings correlated with the cell proliferation studies and demonstrated increased enhancement of radiation-induced apoptosis with the combination of cetuximab/JAK1i compared to the use of either agent alone.

**Fig. 6:**
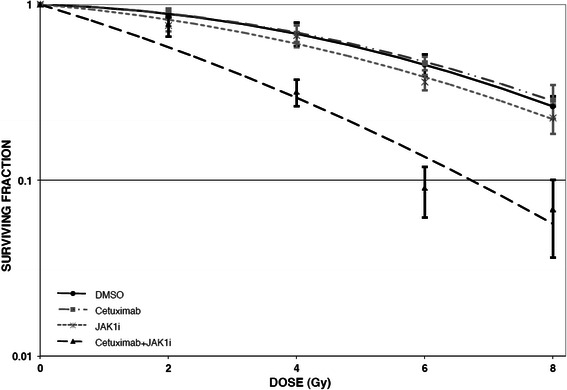
The use of JAK1i (1 μM) combined with cetuximab (0.5 μg/ml) prior to radiation resulted in marked radiosensitization in UM-SCC-1 cells. JAK1i alone or cetuximab alone did not result in radiosensitization. The data represent the mean and standard error of three assessments

Following the studies of cellular proliferation and apoptosis, it was important to assess cell survival. A standard colony formation assay was used for these studies. A representative experiment for UM-SCC-1 cells is shown (Fig. 6). UM-SCC-1 cells were exposed to either JAK1i alone (1 μM), cetuximab (0.5 μg/ml) alone or the combination of JAK1i and cetuximab just prior to radiation. The combination of JAK1i and cetuximab resulted in marked radiosensitization whereas exposure to either agent alone did not result in radiosensitization under these conditions (Fig. 6).

**Fig. 7:**
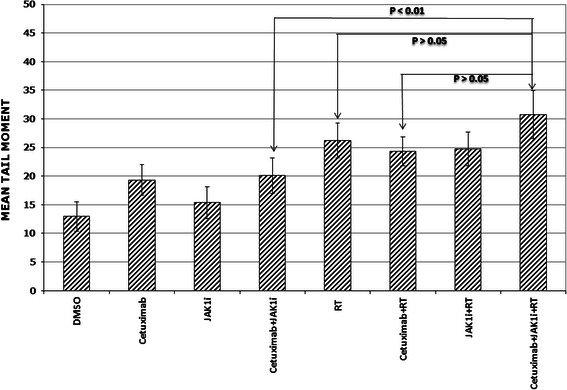
The addition of cetuximab, JAK1i or the combination of cetuixmab and JAK1i prior to radiation did not enhance the number of radiation-induced DNA double strand breaks. UM-SCC-1 cells were treated with cetuximab (0.5 μg/ml) for one hour with and without JAK1i (1 μM) added 5 mins prior to radiation (2 Gy) and subsequently assessed for the mean tail moment as measured by the neutral comet assay and described in the methods section

Since the cytotoxic effects of radiation are associated with the induction of DNA double strand breaks (dsbs), studies were performed to determine whether the addition of JAK1i to cetuximab and radiation resulted in greater induction of DNA dsbs as assessed by the neutral comet assay. A representative example of these studies for the UM-SCC-1 cells is shown in Fig. 7. UM-SCC-1 cells were exposed to cetuximab (0.5 μg/ml) for one hour prior to radiation with and without JAK1i (1 μM) for 5 mins prior to radiation. The induction of DNA dsbs was similar for radiation alone, radiation with either JAK1i or cetuximab and radiation with both JAK1i and cetuximab (Fig. 7). Therefore, the addition of one or both of the two inhibitors did not affect the induction of DNA dsbs.

**Fig. 8:**
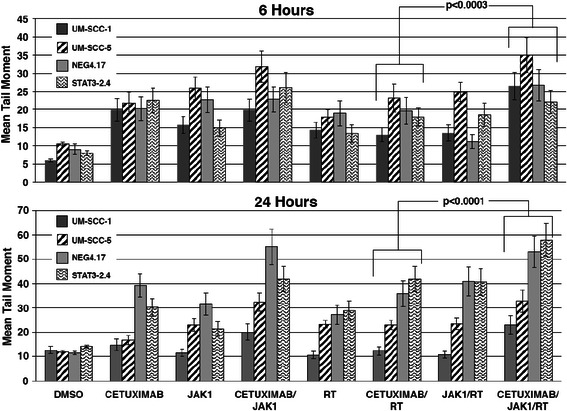
The addition of JAK1i (1 μM) resulted in less repair of radiation-induced DNA dsbs at 6 and 24 h following radiation (2 Gy) compared to cetuximab (0.5 μg/ml) and radiation without JAK1i as measured by the neutral comet assay in all four cell lines. *Top*. The mean tail moment for 6 h of treatments alone or proceeded by radiation. *Bottom*. The mean tail moment for 24 h. Following treatment, exponentially growing cells were collected and processed for single-cell gel electrophoresis assay (Trevigen’s Comet Assay)

Since cetuximab-induced radiosensitization is associated with the inhibition of DNA dsb repair [8, 11], studies were performed to determine if the addition of JAK1i to cetuximab and radiation accentuated this effect. The assessment of DNA dsb repair by the neutral comet assay revealed that the addition of JAK1i to cetuximab resulted in more pronounced inhibition of the repair of DNA damage compared to cetuximab alone in all four cell lines (Fig. 8). Furthermore, the combination of cetuximab and JAK1i resulted in more pronounced inhibition of radiation-induced DNA dsb repair compared to cetuximab-mediated or JAK1i-mediated inhibition of radiation-induced DNA repair (Fig. 8). This effect was demonstrated at 6 and 24 h of repair time in all four cell lines. Taken together, these results suggest that DNA repair is potentially mechanistically involved in the increase in radiosensitization when JAK1i is added to cetuximab and radiation.

## Discussion

Human head and neck squamous cell carcinoma cancer cells show high levels of EGFr expression and it is believed that this expression plays an important role in the EGFr-mediated receptor tyrosine kinase signaling that controls cellular proliferation and can lead to the aggressive growth of these tumors [1]. Increased expression of EGFr has correlated with a poor prognosis for patients with head and neck cancer treated with conventional therapies [12]. The significance of EGFr in the growth of head and neck malignancies led many investigators to explore the use of anti-EGFr treatments in this disease. These efforts proved fruitful as anti-EGFr treatments have been effective as single agent therapies or in combination with chemotherapy for patients with metastatic disease [2, 4]. Additionally, the inhibition of EGFr has resulted in clinically significant radiosensitization for locoregionally advanced tumors [3].

Even though anti-EGFr treatments, such as cetuximab are effective in head and neck cancer, it is known that the EGFr pathway receives additional signaling input from other parallel growth pathways [13]. It is believed that these parallel growth pathways can partially circumvent the anti-proliferative effects of anti-EGFr agents, such as cetuximab, by activating proteins that are downstream of EGFr. Therefore, we sought to enhance the anti-proliferative and radiosensitizing effects of cetuximab by adding an inhibitor of STAT-3 which is an important downstream tyrosine kinase that facilitates protection from apoptosis. Others have targeted STAT-3 in human tumor models using various techniques. Grandis, *et al.*, stably transfected human head and neck cancer cells with dominant negative mutant STAT-3 constructs. This alteration resulted in these constructs failing to proliferate [14]. In contrast, a similar dominant negative construct for STAT-1 did not alter the proliferation of the cells.

Recently, this same group, Sen *et al.* [15], further assessed the effect of targeting STAT-3, utilizing decoy nucleotide sequences that interfere with STAT-3 mediated DNA binding, on the growth rate of cetuximab-sensitive parental T24 squamous cell cancer cells and T24 cells with acquired resistance to cetuximab. The targeting of STAT-3 decreased proliferation in both the cetuximab sensitive and cetuximab resistant cells. These cells were then grown as xenografts and the combination of cetuximab and STAT-3 decoy was assessed. Treatment of either cetuximab-sensitive or cetuximab-resistant cells with cetuximab and STAT-3 decoy resulted in significant reductions in tumor volume compared to cetuximab with a control mutant decoy. They also showed that STAT-3 levels were increased in squamous cell tumors (patient samples) that recurred following cetuximab treatment. Due to the above findings, they hypothesized that the application of STAT-3 inhibitors may be useful in circumventing cetuximab resistance. They did not examine the use of radiation or cetuximab-induced radiosensitization in these experiments.

The work of Sen *et al.*, may serve as appropriate preliminary preclinical data to consider combining inhibitors of EGFr and STAT-3 for incurable tumors [15]. However, adding JAK1i to the combination of cetuximab and radiation could potentially enhance the cure rate of cetuximab and radiation for patients with locoregionally advanced disease. In fact, cetuximab is a known radiosensitizer and only partially inhibits the activation of STAT-3 and hence, we employed a combination of cetuximab and a JAK1-STAT-3 inhibitor (JAK1i) with or without radiation. This approach showed that the two agents interacted to enhance the inhibition of cellular proliferation and promote apoptosis compared to either agent alone. These results corroborated the results of Sen *et al.* [15]. Furthermore, the addition of JAK1i enhanced the radiosensitizing effects of cetuximab in human head and neck cancer cells. Finally, our results showed that the combination of the two agents inhibited radiation-induced DNA repair to a greater extent than either agent alone. These effects were demonstrated in four human head and neck squamous cell cancer cell lines including STAT-3-2.4 cells which showed 50 % STAT-3 knockdown as a result of stable transfection of a short hairpin RNA against STAT-3. This latter result increases the potential ramifications of this study. It is very possible that this dual approach to the inhibition of EGFr signaling (that includes anti-STAT-3) may be clinically relevant for tumors with low to high levels of STAT-3 activation.

The findings that the addition of JAK1i to cetuximab and radiation enhanced the anti-proliferative, apoptotic and radiosensitizing effects of radiation and the accompanying result that there was no appreciable difference in this effect for STAT-3 knockdown cells *vs*. parental cells, may not have been expected. One might have expected JAK1i to be less of a radiosensitizer or add less to the cetuximab-radiation interaction in the STAT-3 knockdown cells. However, this was not the case. These results suggest that even though STAT-3 expression may be reduced in a particular cell line, STAT-3 remains a significant partner with EGFr (and other receptors) regarding the sensitivity of the cell to radiation. It will also be important to investigate whether STAT-3 is a more important driver of EGFr signaling in cell lines with certain molecular characteristics. This information could help investigators exploit STAT-3 inhibition as a clinical target for personalized medicine approaches. Our study did not provide information in this regard. The STAT-3 knockdown cells and parental cells showed similar effects for the studied conditions.

The aforementioned studies were performed based on previous work that demonstrated cetuximab radiosensitized human head and neck squamous cell cancers [1]. The previous findings that cetuximab was a radiosensitizer for squamous cell carcinomas initiated many additional laboratory and clinical investigations in the early 2000’s. Our group reported that cetuximab enhanced radiation-induced apoptosis in human squamous cell cancer, but we did not explore the induction or repair of radiation-induced DNA damage in those early studies. However, following the demonstration that cetuximab was clinically useful as a radiosensitizer [3], others reported that cetuximab inhibited radiation-induced DNA dsb repair [11]. Although cetuximab alters many signaling pathways that could affect radiosensitivity, the potential role of DNA repair in cetuximab-induced radiosensitization for certain cell populations led to the DNA repair studies reported herein. The findings that the addition of JAK1i to cetuximab and radiation resulted in greater radiosensitization and greater slowing of the repair of radiation-induced DNA dsbs are intriguing. Cetuximab’s known effect of slowing the repair of radiation-induced DNA dsbs is hypothesized to be involved in at least a portion of cetuximab-induced radiosensitization in many cell lines. Cetuximab also influences cellular processes such as cell cycle, apoptosis, and angiogenesis. It is likely that these effects play a role in cetuximab-induced radiosensitization as well [16, 17]. As with cetuximab, it is entirely possible that JAK1i creates many effects (due to altered signaling of several pathways) that are involved in radiosensitivity, but our results suggest that greater slowing of the repair of radiation-induced DNA dsbs contributes to the JAK1i - mediated enhancement of cetuximab-induced radiosensitization.

Others have explored the dual inhibition of molecular targets as a method of enhancing the effects of radiation in head and neck cancer by targeting various aspects of EGFr signaling. It is known that EGFr signaling promotes angiogenesis [18]. Cetuximab leads to a partial decrease in angiogenesis for human head and neck cancer [18]. (This partial cetuximab-induced inhibition is similar to cetuximab’s effect on STAT-3). It is believed that cetuximab-mediated inhibition of EGFr leads to partial reduction in angiogenesis through downregulation of HIF-1a and Notch 1 [18]. Since preclinical studies have shown that cetuximab partially inhibits angiogenesis and this inhibition appears to hinder tumor growth, investigators have been interested in combining cetuximab with anti-VEGF agents, such as, bevacizumab. Additionally, there has been interest in combining both agents with radiation in head and neck cancer. Recently, the Memorial Sloan Kettering group treated 30 patients with locoregionally advanced head and neck cancer with cetuximab (400 mg/m^2^ loading dose, followed by 250 mg/ml weekly), bevacizumab (15 mg/kg, day 1 and 22) and cisplatin (50 mg/m^2^, day 1, 2, 22 and 23) concomitantly with radiotherapy (70 Gy). The early results have been promising with an overall survival of 92.8 % at 2 years [19]. Further work will be necessary to determine if the use of this particular combination of dual inhibitors of molecular targets, for the purpose of enhancing radiosensitization, is an advance in the clinical arena.

Additional investigations have employed dual radiosensitizers in head and neck cancer by combining cisplatin with targeted agents. (It is noteworthy that reduced cisplatin doses were employed in the above noted clinical trial with cetuximab, bevacizumab and radiation). Cisplatin has long been used as a radiosensitizer in head and neck cancers. After cetuximab was found to be a radiosensitizer for head and neck cancer, interest arose in combining cisplatin and cetuximab as dual radiosensitizers. The Radiation Therapy Oncology Group (RTOG) recently reported the results of a large phase III study that explored the use of cisplatin-radiotherapy with and without cetuximab for patients with locoregionally advanced head and neck cancer. Unfortunately, the addition of cetuximab to the combination of cisplatin and radiotherapy did not result in a benefit for patients [20]. In the future, combinations of targeted agents with radiotherapy could prove to be an area of impactful investigation as there may be complementary targeted agents that enhance tumor kill without significantly increasing toxicity.

## Conclusion

Our work and the work of others suggest that combinations of agents that target overlapping signal transduction pathways may be a means to address relative resistance to single agents [21]. The present study suggests that the combination of inhibitors of EGFr and STAT-3 enhances the effects of radiation in head and neck cancer compared to the use of either agent alone. Inhibitors of STAT-3 are being investigated [22] and these agents could be combined with cetuximab in future clinical trials.
